# PCR-Based Molecular Characterization of *Toxocara* spp. Using Feces of Stray Cats: A Study from Southwest Iran

**DOI:** 10.1371/journal.pone.0065293

**Published:** 2013-06-03

**Authors:** Shahram Khademvatan, Fakher Rahim, Mahdi Tavalla, Rahman Abdizadeh, Mahmoud Hashemitabar

**Affiliations:** 1 Cellular and Molecular Research Center, Ahvaz Jundishapur University of Medical Sciences, Ahvaz, Iran; 2 Department of Medical Parasitology, Ahvaz Jundishapur University of Medical Sciences, Ahvaz, Iran; 3 Toxicology Research Center, Ahvaz Jundishapur University of Medical Sciences, Ahvaz, Iran; Washington State University, United States of America

## Abstract

Feces of stray cat are potential sources of gastrointestinal parasites and play a crucial role in spreading and transmitting parasite eggs, larvae, and oocysts through contamination of soil, food, or water. In this study, we investigated the prevalence of *Toxocara* spp. infection in stray cats in Ahvaz city, southwest Iran. Eggs of *Toxocara* spp. in feces of stray cats were detected by the sucrose flotation method, and identification was conducted by polymerase chain reaction (PCR) and DNA sequencing. Of the 140 fecal samples that were randomly collected from public environments during the months of January to May 2012, 45% were found to harbour *Toxocara* spp. eggs. The highest prevalence of *Toxocara* spp. eggs was found in the central area of Ahvaz city (28.6%). *T. canis eggs* were found in 4 (6.34%) of the 63 positive samples. Stray cats are found in parks, playgrounds, and other public places and may be a potential contamination risk. Identification of *Toxocara* spp. using molecular methods is sufficiently sensitive to detect low levels of parasites and identify the different *Toxocara* spp. in feces. The relatively high prevalence of *Toxocara* spp. infection may continue to increase due to lack of effective environmental hygiene control in Iran. Consequently, there is a need to plan adequate programs to detect, identify, and control this infection as well as stray cats in the region.

## Introduction

Ascaridida nematodes, such as *Toxocara canis*, *Toxocara cati*, *Toxocara malaysiensis*, and *Toxascaris leonina,* are the most common zoonotic gastrointestinal helminths infecting predatory mammals belonging to Canidae and Felidae families [1]–[4]. An infected dog or cat excretes a huge number of eggs of *Toxocara* spp. into the environment every day. These parasites infect other mammals as definitive hosts, including rodents and humans [5]. Infection occurs through infective eggs, earthworms, cockroaches, birds, and rodents that contain larvae in their tissues [4], [6]. Humans are normally infected through accidental ingestion of embryonated eggs from contaminated soil. The clinical manifestations associated with toxocariasis are classified as visceral larva migrans, ocular larva migrans, covert toxocariasis, and neurological toxocariasis [7]–[12].

In many areas, accurate information about the prevalence of *Toxocara* spp. either does not exist or is assessed based on epidemiology of *T. canis* infection in dogs. Nevertheless, differentiation between the eggs of *T. canis* and *T. cati* has not often been attempted; therefore, it is probable that *T. cati* plays a more important role in human toxocariasis than previously suggested [1], [2]. In research based upon coprological examination of fecal samples, parasite eggs are often identified as *Toxocara* spp., rather than classified to the exact species [4]. Although differentiation among *Toxocara* spp. is clinically and epidemiologically important, identification of species within the genus is complicated [13]. According to Uga *et al*. (2000) measurement of egg dimensions was not helpful in the differentiation of *Toxocara* spp. because approximately 90% of the eggs measured were of similar size. Later, it became possible to differentiate eggs of *T*. *canis* from *T. cati* based on their characteristic surface structure identified using scanning electron microscopy [14]. Various studies have demonstrated that the polymerase chain reaction (PCR)-based molecular approach could provide reliable markers for more accurate identification of *Toxocara* spp. [2], [15], [16]. PCR with specific primers can identify the eggs of one *Toxocara* spp. in the feces of a definitive host.

The populations at greatest risk for *Toxocara* infection include toddlers and small children, since they are most likely to be exposed to contaminated soil or sandpits while playing outdoors [17] [18]. In addition, several occupational groups such as gardeners, farmers, construction workers, and veterinarians are at elevated risk due to their contact with heavily contaminated soil [19].

In Iran, cats are often allowed to pass freely in and out of houses as predators of rats. Stray cats feast on rubbish and leftover food countless times and discharge helminth eggs and protozoan cysts into public environments. Because of the close association of cats with humans in urban areas, stray cats are important sources of a variety of zoonotic parasites, including *Toxocara* [20]. Since comprehensive studies about *Toxocara* spp. have received little attention in Iran, the aim of this study was to identify and determine the prevalence of *T. cati* infection in stray cats found in urban areas of Ahvaz city, southwest Iran.

## Materials and Methods

### Study Area, Design, and Population

Ahvaz city, the capital of the Khuzestan Province, is located in southwest Iran (latitude 31°50′N and longitude 49°11′E). It covers an area of more than 200 km^2^, and has a population of 1,080,955 inhabitants. During the summer, Ahvaz city has an extremely hot and humid climate and the temperature ranges between 48 and 50°C. During the winter, Ahvaz city has a warm climate, with light to moderate rain. A total of 140 fecal samples were collected from open public spaces in five regions of Ahvaz city (north, south, east, west, and central) during the months of January to May 2012. The sampling procedure required no specific permissions for all five regions of Ahvaz city, and the field studies did not involve endangered or protected species. Approximately 50 g of cat feces was collected; the unused portions of samples were hygienically discarded.

### Sample Collection and Fecal Analysis

Fecal samples were put in plastic bags and stored at 4°C until processing, which was performed within 24 h. Isolation of the eggs of *Toxocara* spp. from each sample was performed by the sucrose flotation method as previously described [21]. In brief, fecal samples were suspended in distilled water, centrifuged at 1000 rpm for 5 min, and approximately 30 ml distilled water was added to the sediment. The suspensions were layered with 15 ml of sucrose (Merck, Germany), with specific gravity of 1.40, and centrifuged at 800×g for 5 min. The upper layer of the liquid was separated and centrifuged at 1000×g for 5 min. The sediment was then resuspended in 50 ml distilled water and centrifuged at 5000 rpm for 5 min. Finally, the samples were washed twice with distilled water and sediments were examined under light microscopy (10**×** and 40**×**).

### DNA Isolation and PCR

Genomic DNA from the eggs of *Toxocara* spp. was extracted using the QIAamp DNA Mini Kit (Qiagen AG, Hombrechtikon, Switzerland) according to the manufacturer’s instructions. The samples were first subjected to three freeze–thaw cycles, and proteinase K digestion was performed overnight (∼16 h) as suggested by Borecka and Gawor [15] Species-specific oligonucleotide primers were selected from internal transcribed spacer 2 (ITS2) gene sequences that were previously described as *Tcan1* (5′-AGTATGATGGGCGCGCCAAT-3′) and *NC2* (5′-TAGTTTCTTTTCCTCCGCT-3′) for *T. canis* and *Tcat1* (5′-GGAGAAGTAAACTC-3′) and *NC2* for *T. cati* [22].

PCR amplification was performed using 50 µL of reaction mixture containing 250 µM of each deoxynucleotide, 100 pmol of each primer, 50 mM KCl, 10 mM Tris-HCl (pH 9), 3 mM MgCl_2_, 10% dimethyl sulfoxide (DMSO, Sigma), 2 U of Taq DNA polymerase (Fermentas), and 10–15 ng of template DNA. The PCR reaction was conducted using a thermal cycler (MyCycler; Bio-Rad, Hercules, CA) and the amplification cycle consisted of an initial cycle at 94°C for 30 s; followed by 35 cycles of denaturation at 94°C for 60 s, annealing at 58°C for 30 s, and extension at 72°C for 30 s; and a final extension cycle at 72°C for 10 min. Finally, 20 µL of PCR products were run on a 1.8% agarose gel containing ethidium bromide in 1× Tris-borate EDTA buffer along with 1444–80 bp DNA ladder (Fermentas).

### Sequencing Method

In the present study, PCR amplification of the ITS2 region of nuclear ribosomal DNA (rDNA) was used to identify the eggs of *Toxocara* spp. isolated from stray cats. The results were confirmed by DNA sequencing. DNA derived from 20 infected feces was sequenced by MWG (Germany) using the primers employed during PCR. The resulting data were analyzed using Chromas software (http://technelysium.com.au/).

### Evaluation of the ITS2 Motif Specificity using BLAST

The *Toxocara* ITS sequences were used to query the non-redundant GenBank database of the National Center for Biotechnology Information (nr at NCBI) using BLAST. The BLAST output was then analyzed to find only the exact or perfect pairwise matches showing high percent identity scores and low expect (E) values for each species.

### Phylogenetic and Data Analysis

DNA sequences were initially aligned with ClustalW for multiple sequence alignment, using the default gap and extension penalties from this program [23]. Next, the ITS sequences were entered in PAUP* [24] to generate phylograms for each dataset using the methods of Dettman *et al.* [25], [26], i.e., maximum parsimony, NNI branch swapping, and 500 random sequence additions. Three or four locus-combined datasets were made and subjected to weighted parsimony with the weight for each locus calculated as the number of parsimony informative characters for the least variable locus divided by the parsimony informative characters of that locus. Bootstrapping was performed using PAUP* with sequence addition for 1000 replicates. MrBayes 3.1.2 [27], [28] was used to calculate the posterior probabilities of branches. Parameters were typically left at their default settings.

### Statistical Analysis

Statistical analysis was performed by SPSS 16.0 software. Data are expressed as number and percentage. Difference between groups was analyzed by the chi-square test, and *P*≤0.05 was considered significant.

## Results

In this study, all collected feces were processed by the sucrose flotation method, which showed that 63 (45%) of 140 samples were contaminated with the eggs of *Toxocara* spp. Determining the prevalence of *Toxocara* spp. in cats from each of the five regions of the city (north, south, east, west and central) revealed that the highest prevalence was in the central region (28.6%, 95% CI: 20–38.6) and the lowest (14.3%, 95% CI: 8.3–23) was in both the west and south (*P* = 0.026, Table 1 and 2).

**Table 1:** Results of flotation methods for the detection of the eggs of Toxocara in the five regions of Ahvaz city.

		Method of detection		
Area	No. of examinedfecal samples	Positive	%	95% CI
***North***	25 (17.9%)	14	22.2	14–30
***West***	29 (20.7%)	9	14.3	8.3–23
***East***	28(20.0%)	13	20.6	13.4–30
***South***	26 (18.6%)	9	14.3	8.3–23
***Centre***	32 (22.9%)	18	28.6	20–38.6
***Total***	140(100%)	63	100	_

CI, confidence interval.

**Table 2:** Between group comparison with respected *P* values.

Centre	South	East	West	North	Regions
0.369	0.191	0.806	0.191	____	***North***
0.029	1	0.286	____	−0.191	***West***
0.254	0.286	____	0.286	0.806	***East***
0.029	___	0.286	1	0.191	***South***
____	0.029	0.254	0.029	0.369	***Centre***

**Figure 1:**
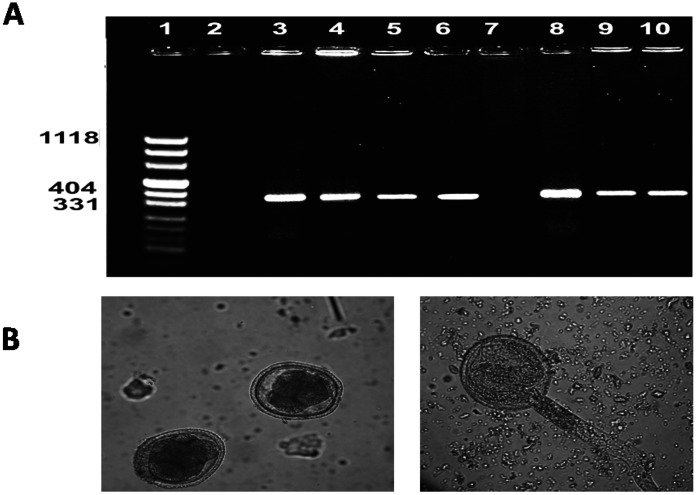
DNA amplification of *Toxocara* spp. isolated from stray cats by PCR on 1.8% agarose gel: lane 1, marker; lane 2 and 7, negative control; lane 3, *T. cati* positive control 370 bp; lane 4, 5 and 6, *T. cati* isolated from feces of cats; lane 8, *T. canis* positive control 380 bp; lane 9 and 10, *T. canis* recovered from feces of cats. (A). Unembryonated (left) and embryonated (right) eggs of *T. cati* from stray cats in Ahvaz city (B).

All *Toxocara*-positive samples were examined by PCR using *Tcan/NC2* and *Tcat/NC2* primers. From 63 positive samples, 96.8% showed 370-bp fragments from *T. cati* and only four showed 380-bp fragments from *T. canis.* However, no mixed infections were observed, and no amplification was observed in the negative controls (Figure 1). Sequence analysis was performed using 16 randomly selected *Tcan/NC2* PCR products and four randomly selected *Tcat/NC2* PCR products. All sequences obtained were submitted to NCBI GenBank; their reference numbers are given in Table 3. GenBank was searched for similar published sequences using BLAST, and significant homology was detected with other *Toxocara* spp. sequences. The sequences of all *Toxocara* spp. isolates were homologous with a small number of differences corresponding to punctual base substitutions (Table 3).

**Table 3:** Sequence analysis of 20 randomly selected eggs of *Toxocara* spp. in the five regions of Ahvaz city.

*Toxocara* spp. identified by PCR	Isolation site	No of submitted sequencein Gene bank	Accession number
	***North***	***4***	AB743610.1, AB743608.1, AB743606.1, AB743604.1
	***West***	***2***	AB743602.1, AB743600.1
***T. cati***	***East***	***3***	AB743598.1, AB743612.1, AB743613.1
	***South***	***2***	AB743611.1, AB743605.1
	***Center***	***5***	AB743603.1, AB743609.1, AB743601.1, AB743599.1, AB743607.1
	***North***	***0***	_
	***West***	***1***	AB743614.1
***T. canis***	***East***	***0***	_
	***South***	***0***	_
	***Center***	***3***	AB743615.1, AB743616.1, AB743617.1
***Total***		***20***	_

**Figure 2:**
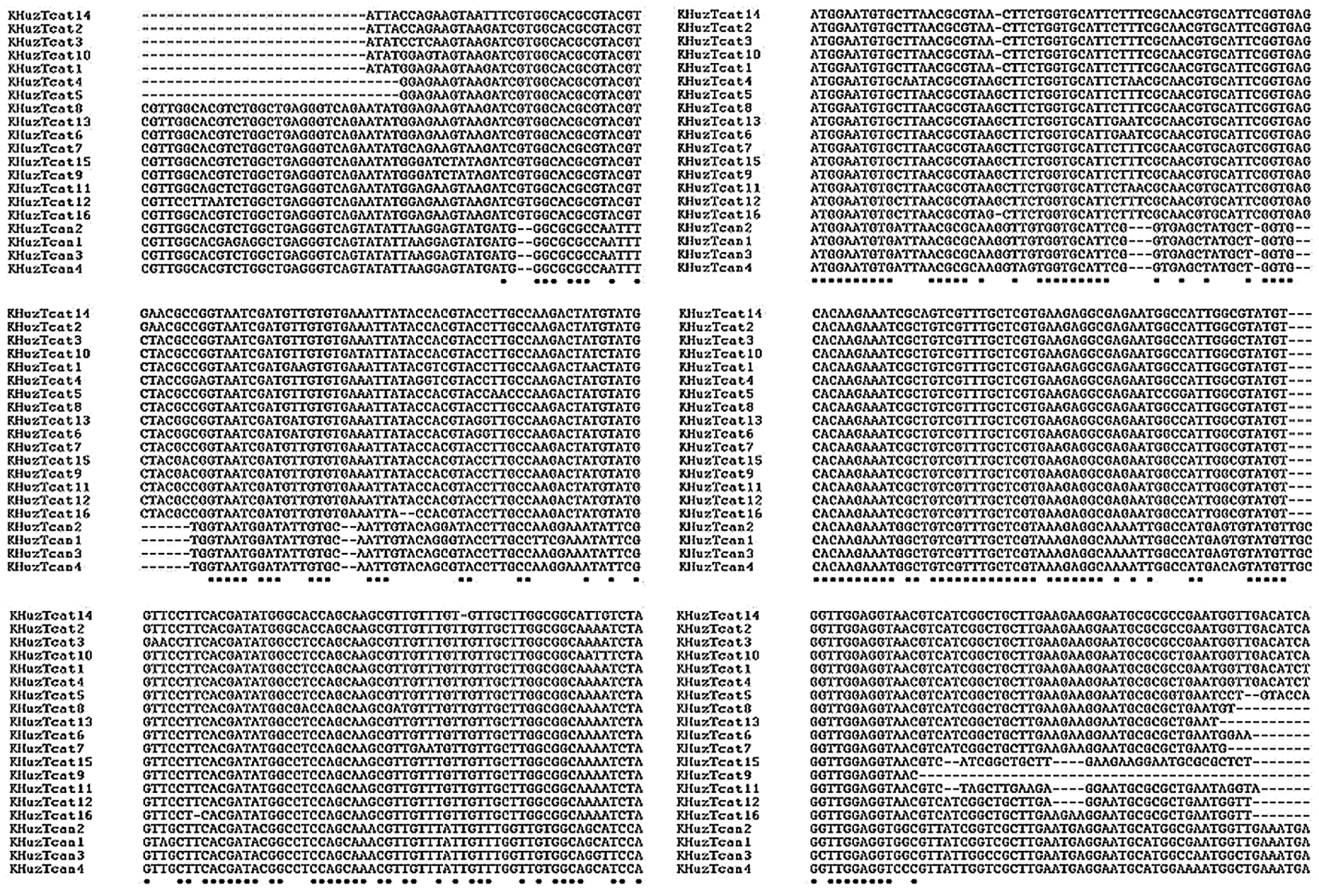
Multiple sequence alignment of the sequences of *Toxocara* spp.

**Figure 3:**
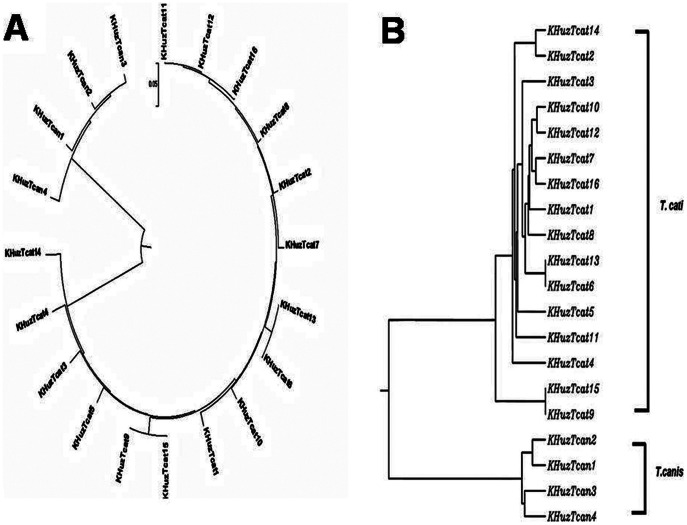
Phylogram derived from combined *T. cati* and *T. canis* data, which strongly support the placement of *T. cati* and *T. canis* in distinct species, although they are supposedly an anamorph/teleomorph pair; (A) Un-rooted tree, (B) Rooted tree.

Multiple sequence alignment revealed conserved nucleotides among the obtained ITS2 sequences (Figure 2). Phylogenetic trees were obtained by comparing our ITS sequences with those of other *Toxocara* spp. Phylogenetic analyses using various distance methods and character methods like maximum parsimony showed that the topology is similar among the trees obtained with significant bootstrap support for the clades. The values of 75% and above in the bootstrap test of phylogenetic accuracy indicate reliable grouping among different members of *Toxocara* spp. (Figure 3).

## Discussion

The present study confirms that stray cats in all areas of the city were infected and that the prevalence of *T. cati* was higher in most localities in Iran than elsewhere in the world. Previous studies have shown various prevalences in *Toxocara* spp. infection in cats in different parts of Iran. The high prevalence of *T. cati* infection found in the present study is similar to the results of a survey from northern Iran (44%) [29]. Arbabi and Hooshyar in central Iran and Sadjjadi *et al.* in southern Iran reported 13.3% and 52.8%, respectively [20], [30]. One study in Turkey showed that the prevalence of *T. cati* was 62.5% [31] whereas other studies in Estonia and Ireland reported a prevalence of 48.2% and 42.8% in adult cats, respectively [32], [33]. It seems that the higher prevalence of *T. cati* in our study is probably because of the climatic conditions, which are suitable for the spread and survival of helminth eggs. Interestingly, our results revealed that the highest prevalence of contamination was in the central region of the city, where we expected lower prevalence of *Toxocara* spp. because of the sanitary and economic conditions prevailing in the city center. The growing number of stray cats and excretion of helminth eggs into the environment were likely factors involved in *T. cati* infections and possible explanations of our findings.

Previous studies conducted morphological tests to differentiate various *Toxocara* spp. via egg size, but it seems that egg size is the least specific criteria used to differentiate between *T. cati* and *T. canis* [14]. PCR-based approaches using the ITS1 and ITS2 segments of rDNA were employed in several studies to differentiate among the eggs of *Toxocara* spp. from adult helminths, which are closely related and/or morphologically similar [2], [15], [16]. Recently, Durant *et al*. used a duplex real-time PCR (2qPCR) method targeting the ITS2 sequences from 53 adult worms to identify the species of the eggs of *Toxocara* spp. in fecal and soil samples. They claimed that the newly developed 2qPCR assay could be considered a useful tool for the detection of the eggs of *T. canis* and/or *T. cati* in fecal samples as well as in soil samples [34].

In this study, PCR with ITS2 primers was used for the identification of the eggs of *Toxocara* spp. isolated form stray cats; the results were confirmed by sequencing. The results of the present study indicate that only four (6.34%) of 63 cat feces samples were contaminated with *T. canis*; DNA sequencing confirms these findings. BLAST analysis at NCBI revealed remarkable homology between our DNA sequence data and the *T. canis* sequences previously submitted to GenBank with accession numbers Y09489 and AB110034 [35], [36]. The phylogenetic studies presented here confirmed that the variation between the sequences obtained herein resulted because they belonged to two different *Toxocara* spp. The natural and experimental establishment of *T. canis* adult infections in the intestines of cats was reported previously [37], [38]. Fahrion *et al.* showed that dogs shed considerable amounts of *T. cati* eggs, but no *T. canis* eggs have been identified in the feces of cats, because coprophagy is common in dogs, and is usually not observed in cats. They mentioned that true host switching of *Toxocara* spp. might be an exceptional event, but their attempts to reproduce patent infections by experimental inoculations of cats with *T. canis* eggs failed [39]. Our results revealed that the highest contamination rate was in the central region of the city, and also three out of four *T. canis* eggs isolated from feces of cats were found in this region. The growing numbers of stray dogs that tend to discharge their feces into these environments could be an important factor in *T. canis* infections of cats, and a possible explanation of this finding.

In conclusion, *Toxocara* spp. can be considered neglected zoonoses, which often occur in areas with poor hygiene and education levels. This study is the first report on the prevalence and identification of the eggs of *Toxocara* spp. isolated from stray cats using molecular methods in the southwestern region of Iran. Differentiation of *Toxocara* spp. using molecular methods such as PCR are sufficiently sensitive to detect low levels of parasites and can identify the eggs of *Toxocara* spp. in feces. There are many stray cats in various parts of the city, which leads to the presence of *Toxocara* infection that may be consider a potential environmental contamination risk for the parks, playgrounds, and other public places.

The relatively high prevalence of *Toxocara* spp. infection may continue to increase due to a lack of effective environmental hygiene control in Iran. Consequently, there is a need to plan adequate programs to detect, differentiate and control such infection, as well as stray cats in the region.
